# Absence of amplification of the FGFR1-gene in human malignant mesothelioma of the pleura: a pilot study

**DOI:** 10.1186/1756-0500-7-549

**Published:** 2014-08-19

**Authors:** Till Plönes, Frank Beckers, Walburga Engel-Riedel, Erich Stoelben, Michael Brockmann, Verena Schildgen, Oliver Schildgen

**Affiliations:** Department of Thoracic Surgery, University Medical Center Witten/Herdecke, Lung Clinic Merheim, Campus Cologne, Ostmerheimerstrasse 200, 51109 Köln, Germany; Institut für Pathologie, Kliniken der Stadt Köln gGmbH, Köln, University Medical Center Witten/Herdecke Merheim, Campus Cologne, Köln, Germany

**Keywords:** Personal medicine, Pleural, Asbestos, Pleuropneumonectomy, Decortication, Chemotherapy

## Abstract

**Background:**

Mesothelioma (MPM) is a rare malignant disease with a worse outcome. Fibroblast growth factor 1 (FGFR1) may be an interesting target for selective tyrosine kinases inhibitors (TKI) in MPM. The aim of this study was to evaluate the amplification of the FGFR1 gene in patients suffering from MPM.

**Findings:**

We identified nineteen male patients treated in our department between August 2008 and July 2010 matching the inclusion criteria. Mean age was 68 years. Histopathological examination confirmed thirteen patients with epitheloid subtype, five with biphasic and one patient with sarcomatoid. Fluorescence in situ hybridization analysis revealed no polysomy nor an amplification of the FGFR gene copy number in any case.

**Conclusion:**

Regarding that also EGFR amplifications in MPM are absolute rarities, our findings may be a hint that TKI’s will not satisfy the hope for a new era in the treatment of MPM.

The malignant pleural mesothelioma (MPM) is an asbestos related highly aggressive malignancy arising from the pleural surface [1]. Despite all advances in diagnostic procedures and therapeutic options, which were made over the last decades, prognosis of MPM is still poor [2]. Most therapeutic strategies like common systemic chemotherapy can improve the overall survival for only a few months. However, alterations in receptor tyrosine kinases (RTK’s) like over expression, mutation or amplification of has been identified as targets for patient-tailored therapy in a broad spectrum of malignancies. In particular, “driver mutations”, like epidermal growth factor receptor (EGFR), anaplastic lymphoma kinase (ALK) and others, are major players involved in the process of carcinogenesis. Physiologically regulating proliferation, apoptosis and cell motility, gene amplification and subsequent over expression of these genes lead to relevant activation of pro-oncogenic pathways and correlates with response to tyrosine kinases inhibitors (TKI’s). It has recently been suggested that the fibroblast growth factor 1 (FGFR1) may be an interesting target for selective TKI’s in MPM [3]. Amplification of FGFR1 is already identified as a therapeutic target in lung cancer, but up to now the role of FGFR1 in mesothelioma is unclear [4].

The aim of this study was to evaluate the amplification of the FGFR1 gene in patients suffering from MPM.

In this retrospective study we investigated nineteen patients suffering from histologically proofed MPM. Histology was confirmed by thoracoscopy and classified according to the World Health Organization (WHO) criteria. All patients were treated in our department between 2008 and 2010. Patients were selected based on the following criteria: no chemotherapy prior tissue sampling, no other malignancy in the medical history and sufficient tissue sampling from the primary tumour. Clinical information was obtained by medical records and our medical data base. The study was approved by the local ethics committee (ethics committee of University of Witten/Herdecke, Nr. 126/2013). Gene copy number analysis of FGFR1 gene was investigated by FISH assay (Cytovision, Berlin, Germany) according to the manufactures instructions. FISH signals were analysed by microscopy performed by experienced molecular biologists blinded to the clinical parameters of each patient. FISH signals were classified in analogy to the HER-2/neu scoring recommendations and counted as amplification if a ratio of >2.2 was observed, whereas a ration <1.8 was counted a negative or not amplified. Due to the descriptive character of this study no further statistic methodology was used.

We identified nineteen male patients treated in our department between August 2008 and July 2010 matching the inclusion criteria. Mean age was 68 years. Histopathological examination confirmed thirteen patients with epitheloid subtype, five with biphasic and one patient with sarcomatoid. Ten patients disclosed exposure to asbestos, which was not confirmed histologically in any case (Table 1). Fluorescence in situ hybridization analysis revealed no polysomy nor an amplification of the FGFR gene copy number in any case (Figure 1).

**Table 1:** **Patients characteristics**

	Value n=19
Age (years) (mean, standard derivation)	68±9
Gender	Male n=19
Asbestos exposure^*^	n=11
Affected lung	left n=5
	right n=13
Histotype	epitheloid n=20
	biphasic n=4
	sarcomatoid n=1

^*^Anamnestic.

**Figure 1:**
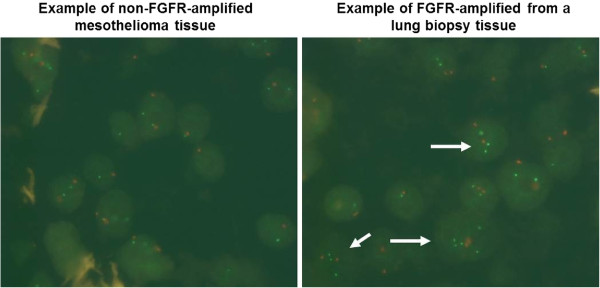
**The figure shows a representative mesothelioma section (5 μm) from FFPE tissue FISH stained with the FGFR probes from Zytovision, Germany (left panel).** No amplification of FGFR (green signals) was observed. As control, a lung section with amplification of the FGFR gene is shown. The white arrows indicate cells in which the green signals are amplified in relation to the chromosal centromer controls (right panel).

## Findings

The MPM is a highly aggressive malignancy, withstanding all current oncological treatment. The inhibition of RTK’s may be a new treatment option in many malignancies and may be an opportunity in the frustrating treatment of MPM. The epidermal growth factor receptor (EGFR) for example is over expressed in many epithelial malignancies, against which TKI’s has been developed. Although EGFR mutations are very rare, the expression status of EGFR in MPM has been noted in several publications [5]. The role of EGFR in MPM is until now unclear, but the fact that MPM is a mesenchymal and not an epithelial tumor may be a reason why the role of EGFR in MPM will remain limited. Therefore other RTK’s has to be evaluated for treatment of MPM. Recently, FGFR1 has been identified as a potential therapeutic target for MPM in an experimental setting [3]. We examined in a pilot study nineteen tissue samples of patients suffering from MPM for amplification of FGFR1-gene. We could not discover any polysomy for FGFR gene in the investigated tissue samples, neither high nor low polysomy. Regarding that also EGFR amplifications in MPM are absolute rarities, this may be a hint that TKI’s will not satisfy the hope for a new era in the treatment of MPM [6, 7]. Further investigations concerning the role of RTK’s and TKI’s in MPM are urgently needed.

### Ethic’s statement

The study was approved by the local ethics committee (ethics committee of University of Witten/Herdecke, Nr. 126/2013).

### Consent section

Written informed consent was not obtained from the patients according to the decisions of the ethics committee of University of Witten/Herdecke.
